# Amikacin Liposome Inhalation Suspension (ALIS) Penetrates Non-tuberculous Mycobacterial Biofilms and Enhances Amikacin Uptake Into Macrophages

**DOI:** 10.3389/fmicb.2018.00915

**Published:** 2018-05-16

**Authors:** Jimin Zhang, Franziska Leifer, Sasha Rose, Dung Yu Chun, Jill Thaisz, Tracey Herr, Mary Nashed, Jayanthi Joseph, Walter R. Perkins, Keith DiPetrillo

**Affiliations:** ^1^Insmed Incorporated, Bridgewater, NJ, United States; ^2^Department of Biomedical Sciences, College of Veterinary Medicine, Oregon State University, Corvallis, OR, United States

**Keywords:** non-tuberculous mycobacteria, amikacin, biofilm, macrophage uptake, ALIS, LAI, Arikayce, Arikace

## Abstract

Non-tuberculous mycobacteria (NTM) cause pulmonary infections in patients with structural lung damage, impaired immunity, or other risk factors. Delivering antibiotics to the sites of these infections is a major hurdle of therapy because pulmonary NTM infections can persist in biofilms or as intracellular infections within macrophages. Inhaled treatments can improve antibiotic delivery into the lungs, but efficient nebulization delivery, distribution throughout the lungs, and penetration into biofilms and macrophages are considerable challenges for this approach. Therefore, we developed amikacin liposome inhalation suspension (ALIS) to overcome these challenges. Nebulization of ALIS has been shown to provide particles within the respirable size range that distribute to both central and peripheral lung compartments in humans. The *in vitro* and *in vivo* efficacy of ALIS against NTM has been demonstrated previously. The key mechanistic questions are whether ALIS penetrates NTM biofilms and enhances amikacin uptake into macrophages. We found that ALIS effectively penetrated throughout NTM biofilms and concentration-dependently reduced the number of viable mycobacteria. Additionally, we found that ALIS improved amikacin uptake by ∼4-fold into cultured macrophages compared with free amikacin. In rats, inhaled ALIS increased amikacin concentrations in pulmonary macrophages by 5- to 8-fold at 2, 6, and 24 h post-dose and retained more amikacin at 24 h in airways and lung tissue relative to inhaled free amikacin. Compared to intravenous free amikacin, a standard-of-care therapy for refractory and severe NTM lung disease, ALIS increased the mean area under the concentration-time curve in lung tissue, airways, and macrophages by 42-, 69-, and 274-fold. These data demonstrate that ALIS effectively penetrates NTM biofilms, enhances amikacin uptake into macrophages, both *in vitro* and *in vivo*, and retains amikacin within airways and lung tissue. An ongoing Phase III trial, adding ALIS to guideline based therapy, met its primary endpoint of culture conversion by month 6. ALIS represents a promising new treatment approach for patients with refractory NTM lung disease.

## Introduction

Non-tuberculous mycobacteria (NTM) are commonly found throughout the environment and cause pulmonary infections in patients with structural lung damage, impaired immunity, or other risk factors (Johnson and Odell, 2014; van Ingen and Kuijper, 2014). Lung disease is the most common manifestation of NTM infections (NTM lung disease; NTM-LD) and patients typically present with either nodular bronchiectatic disease or fibrocavitary disease (Johnson and Odell, 2014). Various mycobacterial species can cause NTM-LD, but species from the *Mycobacterium avium* complex (MAC; including *avium*, *intracellulare*, *chimaera*, and others) are the most common (Johnson and Odell, 2014).

Non-tuberculous mycobacteria can persist extracellularly in biofilms or intracellularly within macrophages and other cells in infected hosts. NTM biofilms are evident in both expectorated sputum samples and in alveolar walls of explanted lungs from infected patients (Qvist et al., 2015). Several lines of evidence demonstrate that MAC species can also live as intracellular infections within monocytes and macrophages (Appelberg, 2006; Awuh and Flo, 2017). *In vitro*, several MAC strains grew well (9- to 43-fold increases) inside isolated human peripheral blood monocytes cultured with human serum, but failed to grow extracellularly in the presence of human serum (Nozawa et al., 1984). In mice systemically infected with *M. avium*, the mycobacteria proliferated in spleen, liver, and lung tissues over the course of several months (Frehel et al., 1991). Electron microscopy localized the mycobacteria exclusively within cells, with no extracellular mycobacteria detected in thin tissue sections of livers and spleens (Frehel et al., 1991). Clinical case reports have also described intracellular MAC infections in humans, with *M. avium* detected inside peripheral blood leukocytes and bone marrow aspirate (Graham et al., 1984; Moffie et al., 1989). These findings indicate that MAC species can live and replicate within macrophages. Therefore, delivering effective antibiotic concentrations into NTM biofilms and cells infected with NTM are important components of treatment.

*Mycobacterium avium* complex species are generally sensitive *in vitro* to aminoglycoside antibiotics, such as amikacin (Brown-Elliott et al., 2013; Cowman et al., 2016), that exhibit concentration-dependent bactericidal effects. However, amikacin and other aminoglycoside antibiotics accumulate poorly in cells, which can limit their effectiveness against intracellular infections (Kesavalu et al., 1990). One way to increase intracellular amikacin delivery is to package the antibiotic into liposomes, which are nanometer sized vesicles composed of a phospholipid bilayer membrane surrounding an aqueous interior compartment (Gregoriadis, 1976a). The physical characteristics of liposomes (small size, low toxicity, tissue/cell targeting) and their capability for delayed or triggered release of cargo molecules make them highly useful drug carriers (Gregoriadis, 1976b). In fact, liposome encapsulation has been shown to improve the ability of amikacin to kill intracellular *M. avium* infections in macrophages (Kesavalu et al., 1990). Moreover, intravenous injection of liposome-encapsulated amikacin improved treatment of systemic *M. avium* infections compared to free amikacin (Cynamon et al., 1989; Ehlers et al., 1996; Petersen et al., 1996), reducing mycobacterial counts in liver and spleen at doses 100-fold lower than free amikacin (Ehlers et al., 1996).

Despite the effectiveness of liposome-encapsulated amikacin against disseminated *M. avium* infections, intravenous administration of liposomal amikacin failed to provide long-term benefit against pulmonary infections in mice (Ehlers et al., 1996). NTM-LD is particularly difficult to treat because it requires delivery of high amounts of antibiotics to the lung while keeping systemic concentrations low to avoid toxicities (Olivier et al., 2014). Inhalation delivery of liposomal amikacin directly into the lungs may address this problem, but this approach faces three major delivery hurdles: (1) efficient nebulization delivery of liposomes to the lungs; (2) distribution of the liposomes throughout the lungs; and, (3) penetration into biofilms and macrophages to reach the sites of infection. Therefore, we developed amikacin liposome inhalation suspension (ALIS), also referred to in previous publications as Arikayce, Arikace or liposomal amikacin for inhalation (LAI), to overcome these challenges and improve the treatment of NTM-LD.

To provide efficient nebulization delivery to the lungs, ALIS was designed to have a high drug to lipid ratio and to retain a consistent fraction of amikacin within the liposome during the nebulization process. ALIS liposomes are composed of dipalmitoylphosphatidylcholine (DPPC) and cholesterol at a 2:1 weight ratio, with 70 mg/mL of amikacin and 47 mg/mL of lipid (DPPC and cholesterol combined). The high drug loading is achieved through a proprietary infusion process that mixes a stream of lipids dissolved in ethanol with a stream of amikacin sulfate dissolved in water at a specific flow rate ratio (Li et al., 2008), causing amikacin to be in a semi-soluble, coacervated state during liposome formation. The high drug load within each liposome is critical to deliver an effective dose of amikacin to NTM-LD patients in about 14 min using a product-specific eFlow^®^ nebulizer (Lamira^TM^ Nebulizer System; PARI Respiratory Equipment, Inc.). The liposomes are relatively small (<300 nm in diameter) and release about 30% of the initial amikacin load during nebulization. Approximately 67% of the aerosol droplets produced are within the respirable range (<5.0 μm), which enables 43% of the nominal liposome dose to be deposited in the lungs of NTM-LD patients after nebulization (Olivier et al., 2016).

Once inhaled into the lungs, ALIS must disperse throughout the lungs and penetrate through mucus to reach infected areas. In healthy animals dosed with ALIS by inhalation, amikacin was distributed evenly throughout the lungs after single and multiple doses, with equal amikacin concentrations in all lobes of both lungs (Malinin et al., 2016). Similarly, inhaled ALIS distributed throughout the lungs in both healthy volunteers (Weers et al., 2009) and patients with NTM-LD (Olivier et al., 2016); the ratio of distribution to central and peripheral lung regions was approximately 1.6–2.0 (Weers et al., 2009; Olivier et al., 2016) and more than 50% of the deposited dose was detectable in the lung 24 h post-dose (Olivier et al., 2016). Mucus represents a further barrier to distribution of inhaled particles, but ALIS liposomes effectively diffuse through 500–1000 μm thick human mucus in 30 min (Meers et al., 2008). These studies show that inhaled ALIS can distribute across lung regions and through mucus.

The final delivery challenge for ALIS is reaching sites of infection where NTM reside. Previous work demonstrated *in vitro* efficacy with ALIS against multiple *M. avium* and *M. abscessus* strains in a THP-1 macrophage model of infection (Rose et al., 2014). Furthermore, inhaled, nebulized ALIS reduced the NTM burden in mice with respiratory *M. avium* infections (Rose et al., 2014). However, the mechanistic data demonstrating ALIS penetration into NTM biofilms and macrophages are limited. Therefore, we evaluated a range of ALIS concentrations against *M. avium* biofilms and we also quantified ALIS uptake into macrophages, both *in vitro* and in rats given inhaled ALIS.

## Materials and Methods

### Preparation and Characterization of ALIS Containing TAMRA-Amikacin

Amikacin liposome inhalation suspension was prepared using a microscale flow focusing method. DPPC (NOF America; cat # COATSOME^®^ MC-6060) and cholesterol (Avanti Polar Lipids; cat # 700000) were dissolved at a 2:1 weight ratio in 100% ethanol at 20 mg/mL. Amikacin sulfate was dissolved in sterile, deionized water at 80 mg/mL (53.4 mg/mL amikacin base; pH adjusted to 6.7 using 5 M NaOH). For studies requiring fluorescent liposomes, 0.01% AF647-labeled dipalmitoylphosphatidylethanolamine (DPPE; Avanti Polar Lipids; cat # 850705) was included in the lipid solution and 3% tetramethylrhodamine (TAMRA)-amikacin (AAT Bioquest) was included in the amikacin stock. The lipid and amikacin solutions were infused (17 and 34 mL/min flow rates, respectively) through a Y connector, mixed with 2/3 volumes of 275 mM sodium citrate buffer at pH 8, and the resulting liposome suspension was diluted 1:1 in the collection vial with 1.5% NaCl. Samples were subsequently washed via tangential flow filtration using 2 EMD Millipore Pellicon XL cassettes (500 kDa polyethersulfone membrane; cat # PXB500C50).

Total amikacin concentrations in the liposome suspensions were measured by high-performance liquid chromatography (HPLC) using a Hypersil GOLD C18 column (175 Å, 3 μm, 150 mm × 4.6 mm; Thermo Fisher; cat. # 25003-154630) with a mobile phase of 65% methanol, 35% water, and 0.3% pentafluoropropionic acid (PFPA). An aliquot of each liposome suspension was centrifuged in an Amicon Ultra-0.5 centrifugal filter unit with Ultracel-30 KDa membrane to separate free amikacin, and the amount of free amikacin was then determined by HPLC. The lipid concentrations were measured by HPLC using an XBridge C8 column (130 Å, 3.5 μm, 150 mm × 4.6 mm; Waters; cat. # 186003055) with a mobile phase A consisting of 49.9% acetonitrile, 49.9% water, 0.1% acetic acid, and 0.1% triethylamine and mobile phase B consisting of 44.9% acetonitrile, 45% isopropyl alcohol, 10% water, 0.1% acetic acid, and 0.1% triethylamine. Liposome sizes were determined using a Mobius dynamic light scattering instrument (MOB-131, Wyatt Technology).

To confirm the TAMRA-amikacin concentration in the final sample of fluorescent ALIS, liposomes were dissolved with octylthioglucoside detergent to eliminate any minor-self quenching effect and TAMRA fluorescence was measured on a plate reader (Biotek Synergy NEO; BioTek Instruments, Inc., Winooski, VT, United States). Standards with decreasing percentages of TAMRA-amikacin mixed with amikacin (total amikacin concentration fixed at 80 mg/mL) were measured simultaneously and the final TAMRA-amikacin concentration of 0.44% in the ALIS sample was calculated from the standard curve. The same amount (0.44%) of TAMRA-amikacin was used for the free amikacin sample.

### *Mycobacterium avium* Biofilm Assays

The biofilm studies were performed with the A5 strain of *M. avium* subspecies *hominissuis* that was originally isolated from an AIDS patient with a pulmonary infection. For imaging studies, mycobacteria were cultured for 7 days in 2-well chamber slides to establish biofilms. The biofilms were treated with 512 μg/mL of fluorescently labeled ALIS (AF647) for 4 h, fixed with 4% paraformaldehyde for 15 min, stained with Syto9 for 15 min, and sealed with a coverslip. Biofilms were imaged with a Zeiss LSM 780 confocal scanning microscope (630× magnification) and Zen 2.3 software. For efficacy studies, mycobacteria were cultured on plates containing Middlebrook 7H10 agar supplemented with 10% oleic acid-albumin-dextrose-catalase (OADC; Hardy Diagnostics, Santa Maria, CA, United States) at 37°C for 7–10 days and then resuspended into Hank’s Balanced Salt Solution (HBSS; Corning, Corning, NY, United States) at 3 × 10^8^ CFU/mL using optical density. Input suspensions were serially diluted 10-fold and plated to determine the inoculum for each experiment. The input suspension was added to flat-bottom, 96-well polystyrene plates (150 μL/well) and biofilms were formed statically at room temperature for 7 days. After 7 days, supernatants were removed and replaced with HBSS containing 60 mM lactose ± increasing concentrations of either 100% ALIS or 70% ALIS/30% free amikacin (total amikacin concentrations tested were 16, 32, 64, 128, 256, 512, or 1024 μg/mL). Biofilms were treated for 4 days and then disrupted by pipetting the supernatant 50 times. The mixture was serially diluted 10-fold to a final dilution of 10^4^ and the 10^2^–10^4^ dilutions were spot plated in duplicate to quantify CFU. Three independent experiments were performed on different days, with three technical replicates for each condition tested.

### Macrophage Uptake Assay

THP-1 human peripheral blood monocytes (ATCC; cat. # TIB-202) were seeded in 12-well plates at 1.2 × 10^6^ cells/well in RPMI-1640 media (ATCC; cat. # 30-2001) containing 10% fetal bovine serum (FBS; HyClone; cat. # SH30070.03) and 50 ng/mL phorbol myristate acetate to induce attachment and differentiation into macrophages. After 24 h, the media was replaced with fresh RPMI media containing 5% FBS. After another 24 h, the media was replaced with 1 mL of fresh RPMI media with 5% FBS, and 250 μL of ALIS (0.44% TAMRA-amikacin), free amikacin (0.44% TAMRA-amikacin), or control solution were added to respective wells to achieve the final desired concentrations (8, 16, 32, 64, or 128 μg/mL total amikacin). ALIS and free amikacin stocks were dissolved in 300 mM lactose solution. Following 4- or 24-h incubation periods, the media was removed, and then the cells were washed once with phosphate-buffered saline (PBS) and harvested into a 96-well plate. The cells were centrifuged at 300 ×*g*, washed twice with PBS, and suspended in 200 μL of PBS. A small aliquot of the cell suspension was removed for imaging and the remaining cell suspension was used to quantify TAMRA-amikacin fluorescence using a Guava easyCyte 6HT flow cytometer (Millipore) with Easy Check beads as an internal control. The mean fluorescence intensity (MFI) was measured for 5,000 cells and normalized to the TAMRA fluorescence added to each well to account for any slight differences. The assay was repeated in three independent experiments, and then averages and standard errors of the mean were calculated for each treatment condition.

### Macrophage Imaging

The aliquot removed prior to flow cytometry analysis was transferred to another 96-well plate and fixed in 4% formaldehyde. Fixed cells were mounted to glass slides with ProLong^TM^ Diamond Antifade Mountant with 4′,6-diamidino-2-phenylindole (DAPI; Thermo Fisher; cat. # P36962) and coverslips were sealed with clear nail polish. Samples were imaged with a Zeiss Axio fluorescence microscope (400× magnification) and Zen 2.3 software. Settings were kept constant while imaging all samples from the same timepoint.

### Animals and Housing Conditions

Male, Han-Wistar rats were purchased from Charles River Laboratories, group-housed in plastic cages, and maintained with an average room temperature of 69°F (range 68–78°F), an average room humidity of 49% (range 30–70%), and a 12-h light cycle (light on 6:00–18:00). Animals received standard chow (Purina diet; cat. #5053) and water *ad libitum* throughout the study, except during the nebulization period. This study was carried out in accordance with the recommendations of the Guide for the Care and Use of Laboratory Animals. The protocol was approved by the animal care and use committee at Rutgers University.

### Evaluation of Macrophage Content in Cells Collected by Bronchoalveolar Lavage

Four naïve rats were anesthetized under isoflurane anesthesia and bronchoalveolar lavage (BAL) was performed with 2 mL of PBS. The BAL fluid was centrifuged at 2,000 ×*g*, the resulting cell pellet was resuspended in FACS staining buffer, and Fc receptors were blocked for 30 min at 4°C. Cells were stained for 30 min with an Alexa Fluor 700 anti-rat CD45 antibody (BioLegend, San Diego, CA, United States), an Alexa Fluor 488 mouse anti-rat CD11b antibody (Bio-Rad, Hercules, CA, United States), a mouse anti-rat CD11c antibody (Bio-Rad) pre-conjugated with Cy5, and a biotin-conjugated mouse anti-rat CD68 antibody (Bio-Rad), followed by a phycoerythrin-conjugated streptavidin (BioLegend). Finally, all cell samples were stained with DAPI. Cell populations were analyzed using a BD Influx Cell Sorter (BD Biosciences, San Jose, CA, United States), and flow cytometry compensation setting was performed with UltraComp eBeads^TM^ (Thermo Fisher Scientific, Waltham, MA, United States) and with cell samples. The NR8383 rat alveolar macrophage cell line was used as a positive control for staining and flow cytometry.

### Administration of ALIS and Free Amikacin

For inhalation dosing, rats were placed into plastic tube restrainers and loaded into an ONARES nose-only inhalation delivery system (CH Technologies, Westwood, NJ, United States). Drug suspension/solution (40 mL of ALIS or free amikacin) was split between two Aeroneb Pro nebulizers (Aerogen, Chicago, IL, United States) placed in series along the air flow (3 L/min) into the ONARES chamber. The aerosol concentration in the ONARES chamber was sampled 5 min after the start of nebulization by drawing aerosol through a Pall #61631 glass-fiber filter (MilliporeSigma, St. Louis, MO, United States) at 0.25 L/min for 5 min. The filters were stored at 4°C in a glass vial for subsequent amikacin measurements. Nebulization continued until 40 mL of drug solution/suspension was nebulized and the nebulizers ran clear for one additional minute (total nebulization time was 60–90 min).

The study consisted of three experimental groups: ALIS (40 mL at 53.4 mg/mL amikacin base); low-dose free amikacin (40 mL at 20.0 mg/mL amikacin base); and, high-dose free amikacin (40 mL at 53.4 mg/mL amikacin base). Each timepoint for each experimental group required a separate nebulization group consisting of 10 rats; lungs were collected from 2 rats immediately post-dose at the end of the nebulization period to determine the deposited dose of amikacin, and the remaining 8 rats were euthanized together at one timepoint, either 2, 6, or 24 h post-dose. Therefore, each experimental group consisted of 30 rats total, with *n* = 6 euthanized immediately post-dose to measure deposited doses (2 from each of 3 nebulization groups) and *n* = 8 euthanized at each timepoint of 2, 6, and 24 h.

The distribution of intravenous amikacin into pulmonary macrophages, airways, and lung tissue was also assessed using a similar study design. Ten rats in each group were injected with a 20 mg/mL amikacin solution in 0.9% NaCl at 5 mL/kg to achieve a dose of 100 mg/kg. From each group, 2 animals were euthanized 0.25 h post-dose (similar to the immediately post-dose group for the nebulization studies) and the remaining 8 were euthanized together at either 2, 6, or 24 h post-dose. BAL fluid and cell pellets, lungs, and plasma were collected from each rat as described below.

### Blood, Bronchoalveolar Lavage Fluid, and Lung Tissue Collection

At the appropriate timepoint after dosing, rats were weighed and deeply anesthetized with isoflurane, blood was collected by retroorbital bleeding into ethylenediaminetetraacetic acid (EDTA) coated BD Microtainer^®^ tubes (cat. # 02 669-33; Becton Dickinson, Franklin Lakes, NJ, United States), and then rats were euthanized by exsanguination. The lungs were lavaged with 2 mL of PBS and the BAL fluid was collected. Lungs were excised, weighed, rinsed in PBS, placed in 5 mL Eppendorf tubes, and flash frozen in liquid nitrogen. Blood samples were kept on ice prior to centrifugation at 20,817 ×*g* for 10 min, and then the plasma was collected and stored at -80°C until analysis. BAL fluid was centrifuged at 2,000 ×*g* for 3 min and the supernatant was removed and stored at -80°C. The cell pellet was resuspended in 1 mL of fresh PBS and centrifuged at 2,000 ×*g* for 3 min. The supernatant was discarded and the cell pellet was stored at -80°C until analysis.

### Quantification of Sample Amikacin Concentrations

Amikacin standards were generated in each sample matrix: cultured NR8383 rat alveolar macrophage cells were used for BAL cell pellet standards; PBS was utilized for BAL fluid standards; lung homogenate from naïve rats was employed for lung homogenate standards; and, plasma from naïve rats was used for plasma standards. For each standard curve, a 1.0 mg/mL intermediate in the appropriate matrix was first prepared from a primary amikacin stock of 20.0 mg/mL in 20% methanol and 80% water, then further diluted to 100 μg/mL in the appropriate matrix and serially diluted to make calibration standards. Quality control samples were also prepared from the highest standard. Experimental samples were mixed with an equal volume of internal standard working solution (gentamicin C1 in water) and 10% trichloroacetic acid, vortexed for 5 s, and centrifuged at 21,130 ×*g* at 4°C for 15 min. Depending on the analytical range, the supernatant was mixed with either equal volume or 10× volume of mobile phase A (0.2% formic acid and 0.1% heptafluorobutyric acid in water) and 10 μL was injected into a Phenomenex Kinetex Phenyl-Hexyl column (4.6 mm × 50 mm; 2.6 μm; 50°C; 1 mL/min) with an elution gradient consisting of mobile phase A and mobile phase B (100% acetonitrile). Liquid chromatography, tandem mass spectrometry (LC-MS/MS) was performed using a Sciex API4500 mass spectrometer (AB SCIEX, Framingham, MA, United States).

### Quantification of Protein Concentrations

A portion of rat BAL cell pellet lysate and lung homogenate samples were retained for protein quantification using a Pierce^TM^ BCA Protein Assay Kit (Thermo Fisher) according to the manufacturer’s instructions.

### Statistical Analyses

Statistical analyses were performed with GraphPad Prism (La Jolla, CA, United States). Because the inoculation levels were different between the three independent biofilm experiments (*P* = 0.0007 by one-way ANOVA with Tukey post-test), the CFU count for each concentration was normalized as percent of the untreated CFU count within each experiment and then group means and standard errors were calculated. Significant changes from the untreated control group were identified by one-way ANOVA with Dunnett’s multiple comparisons test. For the *in vitro* macrophage uptake experiments, the data were analyzed by two-way ANOVA with Tukey post-test. For the *in vivo* studies, differences between the dose groups at each timepoint were determined by one-way analysis of variance (ANOVA) with Tukey post-test. *P* < 0.05 was considered significant.

## Results

### ALIS Penetrates and Kills *Mycobacterium avium* Biofilms

**FIGURE 1:**
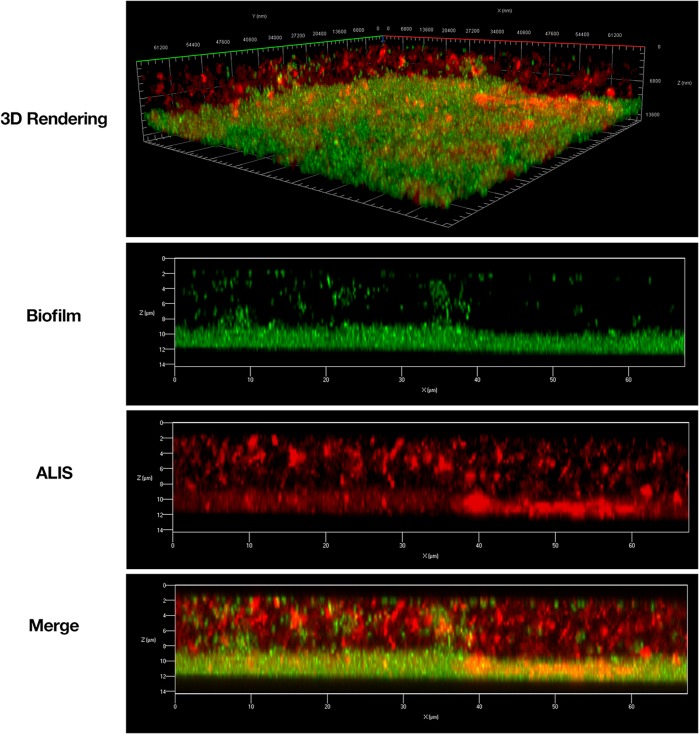
Amikacin liposome inhalation suspension (ALIS) penetrated *Mycobacterium avium* biofilms. Biofilms (strain A5) were established for 7 days in 2-well chamber slides, treated with 512 μg/mL of AF657-labeled ALIS (red) for 4 h, fixed, stained with Syto9 (green), and imaged with a Zeiss LSM 780 confocal scanning microscope (630× magnification).

**FIGURE 2:**
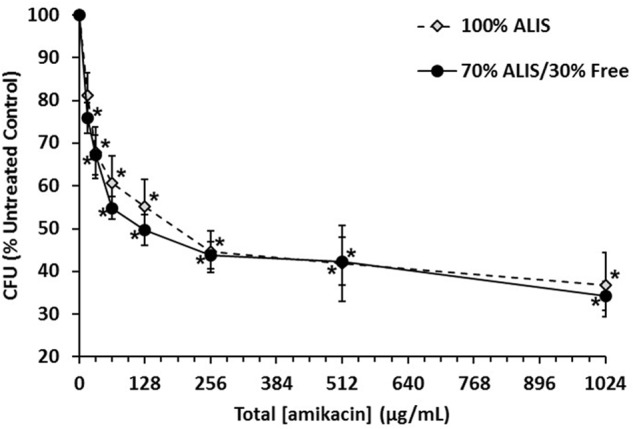
Amikacin liposome inhalation suspension (ALIS) reduced viable cell counts in *Mycobacterium avium* biofilms. Biofilms (strain A5) were established for 7 days in plastic 96-well plates and then treated for 4 days with either 100% ALIS or a 70% ALIS/30% free amikacin mixture that replicates the ratio of liposomal to free amikacin after nebulization. Three independent experiments were performed on different days, with three technical replicates for each condition tested. The colony forming unit (CFU) count for each concentration was normalized as percent of the untreated CFU count within each experiment and then group means and standard errors were calculated. ^∗^*P* < 0.05 compared to no amikacin by one-way ANOVA with Dunnett’s multiple comparisons post-test.

During respiratory NTM infection, one of the niches the bacteria can reside within are biofilms. ALIS is effective at treating NTM infections, but it is unknown if it can penetrate and kill NTM biofilms. We treated *M. avium* biofilms for 4 h with AF647-labeled ALIS to evaluate whether the liposomes can penetrate into the biofilm. Mycobacteria formed a dense biofilm on the slide surface, with more diffuse bacteria and extracellular biofilm components above (**Figure 1**). Liposomes were visible throughout the biofilm, indicating that the liposomes penetrated through the extracellular components and reached the cell dense region (**Figure 1**). We also tested whether increasing concentrations of ALIS can effectively kill the mycobacteria within the biofilms, using both 100% ALIS and a mix of 70% ALIS/30% free amikacin that represents the ratio of liposomal/free amikacin after nebulization. ALIS concentration-dependently reduced viable cell counts in biofilms at concentrations greater than 16 μg/mL, and the 100% ALIS and 70% ALIS/30% free amikacin treatment groups were equally effective (**Figure 2**).

### ALIS Enhanced *in Vitro* Amikacin Uptake Into Macrophages Compared to Free Amikacin

Non-tuberculous mycobacteria are intracellular pathogens that mainly reside within macrophages during infection, creating an additional challenge to deliver effective antibiotic concentrations inside the cell. Taking this important point into consideration, we used fluorescently labeled amikacin to investigate macrophage uptake of ALIS. The fluorescent ALIS formulation produced for uptake studies contained 0.44% TAMRA-amikacin; therefore, we used the same 0.44% TAMRA-amikacin concentration in the free amikacin sample for all experiments to ensure that the total fluorescence added was equal for each comparison between ALIS and free amikacin. The batch of fluorescent ALIS had characteristics similar to a previous engineering batch (ENG1505) of ALIS that represents a typical batch produced by the manufacturing process (**Table 1**).

**Table 1:** Characteristics of fluorescent ALIS batch used in macrophage uptake studies.

Parameter	Fluorescent ALIS	Batch ENG1505^1^
Particle diameter (nm), Nicomp	221 ± 98	321 ± 134
DPPC:Chol (w:w)	1.91	2.06
Drug (amikacin base)/Lipid	0.86	1.38
Amikacin encapsulation	88.3%	100%

*^1^Batch ENG1505 represents a typical batch produced by the ALIS manufacturing process.*

To confirm that TAMRA conjugation did not alter macrophage uptake of amikacin, we performed a pilot study using mass spectrometry to measure the ratio of TAMRA-labeled to unlabeled amikacin in the cell pellet after 24-h incubation with free amikacin or ALIS. Cells treated with either free amikacin or ALIS had the same ratio of unlabeled/TAMRA-labeled amikacin (data not shown).

**FIGURE 3:**
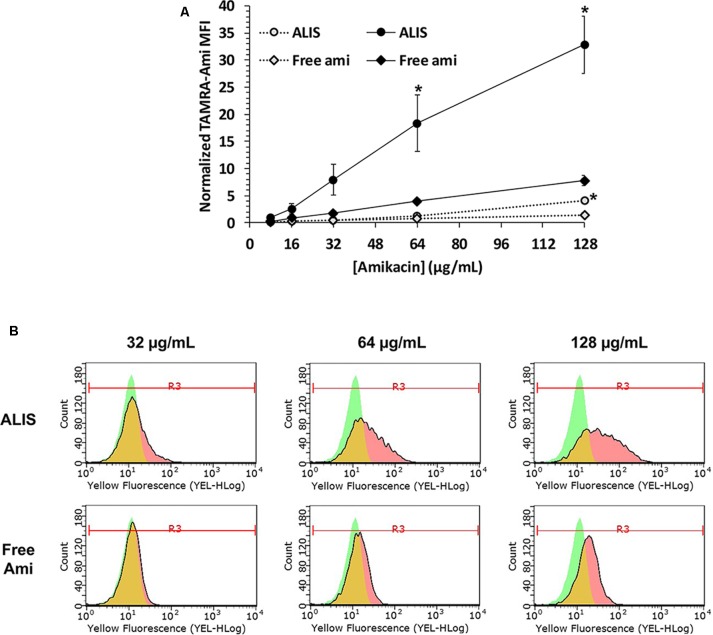
Quantification of liposomal and free amikacin uptake into human macrophages by flow cytometry. ALIS demonstrated higher uptake into differentiated THP-1 macrophages than free amikacin at each concentration. Macrophages were exposed to increasing concentrations of either ALIS or free amikacin (0.44% labeled with TAMRA) for 4 h (gray symbols) or 24 h (black symbols) and, cell monolayers were resuspended, then cellular TAMRA fluorescence was quantified by flow cytometry. Panel **(A)** shows normalized mean fluorescence intensity (MFI) at each concentration averaged from three independent experiments. Panel **(B)** shows representative flow cytometry histograms with gating for the MFI after 24 h treatment from one experiment. Green represents control cells, red represents cells treated with ALIS or free amikacin, and yellow indicates overlap between the two histograms. ^∗^*P* < 0.05 vs. free amikacin at the same concentration and timepoint.

We quantified the uptake of various concentrations of free or liposomal TAMRA-amikacin into human macrophages after incubation for 4 or 24 h. Amikacin uptake was low after 4-h incubation, but cells treated with 128 μg/mL ALIS contained significantly more amikacin than cells treated with the same concentration of free amikacin (**Figure 3A**). After 24-h incubation, macrophages treated with ALIS generally contained ∼4-fold more amikacin than cells exposed to the same concentrations of free amikacin, with significant differences between ALIS and free amikacin groups exposed to 64 and 128 μg/mL concentrations (**Figure 3A**). The flow cytometry histograms demonstrated unimodal distributions for both free and liposomal amikacin at all concentrations tested (**Figure 3B**).

**FIGURE 4:**
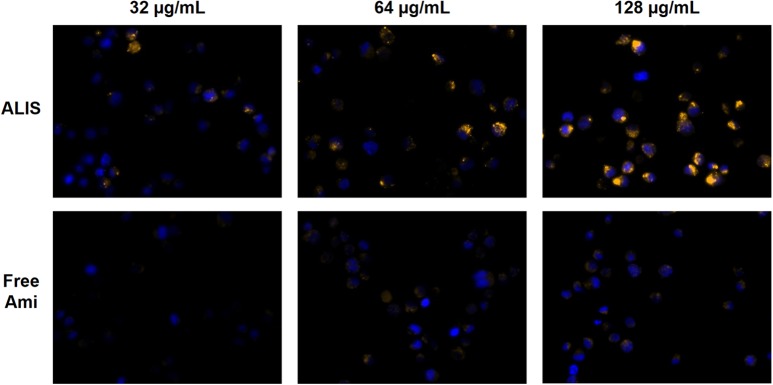
Visualization of liposomal and free amikacin uptake in human macrophages by fluorescence microscopy. ALIS demonstrated higher uptake into differentiated THP-1 macrophages than free amikacin at each concentration. Macrophages were exposed to increasing concentrations of either liposomal or free amikacin (0.44% labeled with TAMRA) for 24 h and then TAMRA fluorescence was visualized by a Zeiss Axio fluorescence microscope (400× magnification) using constant settings for all experimental conditions. Yellow: TAMRA amikacin; Blue: DAPI-stained DNA.

Fluorescence microscopy images taken after 24-h incubation were consistent with the flow cytometry measurements. Macrophages exposed to 32, 64, or 128 μg/mL of ALIS clearly exhibited TAMRA fluorescence, whereas TAMRA fluorescence was sparse and dim in cells incubated with the same concentrations of free amikacin (**Figure 4**).

### ALIS Enhanced *in Vivo* Amikacin Uptake Into Macrophages Compared to Free Amikacin

**FIGURE 5:**
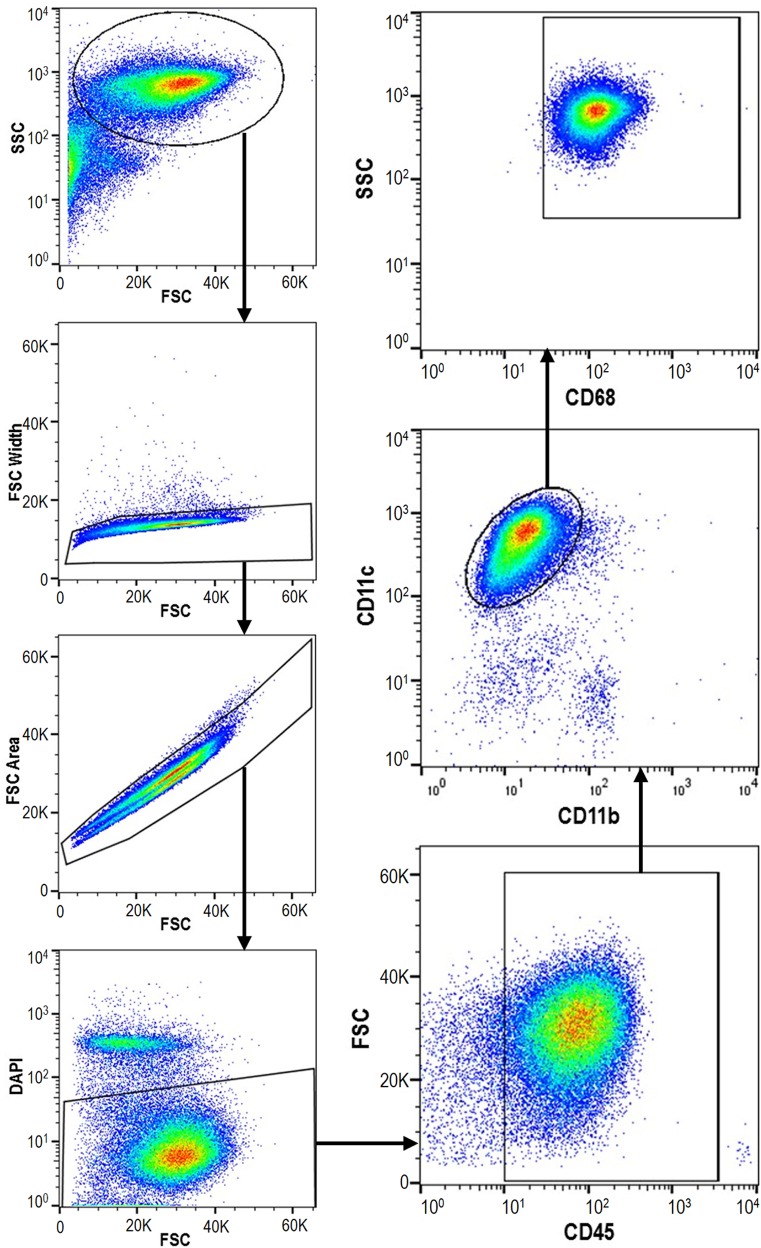
Macrophages represent >80% of cells isolated by bronchoalveolar lavage (BAL). Cells were collected from lungs of naïve rats (*n* = 4) by BAL, labeled with antibodies differentiating macrophages from other leukocytes, and counted by flow cytometry. SSC, side scatter; FSC, forward scatter.

To establish a method to collect pulmonary macrophages, we used flow cytometry to determine the percentage of macrophages among cells collected by BAL from naïve rats. Side and forward scatter differentiated single cells from other debris and cell aggregates in the sample, and high DAPI fluorescence identified dead cells for removal from the analysis (**Figure 5**). CD45^+^ staining identified leukocytes and CD11c^high^/CD11b^low^ staining differentiated alveolar macrophages within the leukocyte population (**Figure 5**). High CD68^+^ staining confirmed the identity of macrophages. Overall, 82.0 ± 3.3% of live cells isolated by BAL were alveolar macrophages.

Because the percentage of macrophages collected by BAL was high, we used total BAL cells for subsequent studies evaluating *in vivo* amikacin uptake into macrophages from rats given either ALIS or free amikacin by nebulization. From each nebulization group, we euthanized two rats immediately post-dose and measured the amikacin dose deposited in the lungs. The amikacin doses delivered to the nose or deposited in the lungs were not significantly different between the ALIS and low-dose amikacin groups (**Table 2**), but high-dose amikacin achieved significantly higher delivered and deposited doses than the other groups (**Table 2**).

**Table 2:** Delivered and deposited amikacin doses in rats administered either ALIS or free amikacin by nebulization.

	ALIS	Amikacin (low dose)	Amikacin (high dose)
Delivered dose (mg/kg BW)	96 ± 4	67 ± 3	161 ± 37^∗^
Deposited dose (μg/g lung)	977 ± 106	507 ± 64	2283 ± 308^∗∗^

*Delivered dose was calculated as the aerosol concentration (calculated from filter samples captured during nebulization) × respiratory minute volume × duration of nebulization/body weight. The deposited dose was measured as the lung amikacin concentration immediately post-dose in two rats from each nebulization group. Values represent mean ± SEM. ^∗^P < 0.05 vs. low-dose amikacin; n = 3 filters per group. ^∗∗^P < 0.05 vs. both low-dose amikacin and ALIS; n = 6 rats per group.*

**FIGURE 6:**
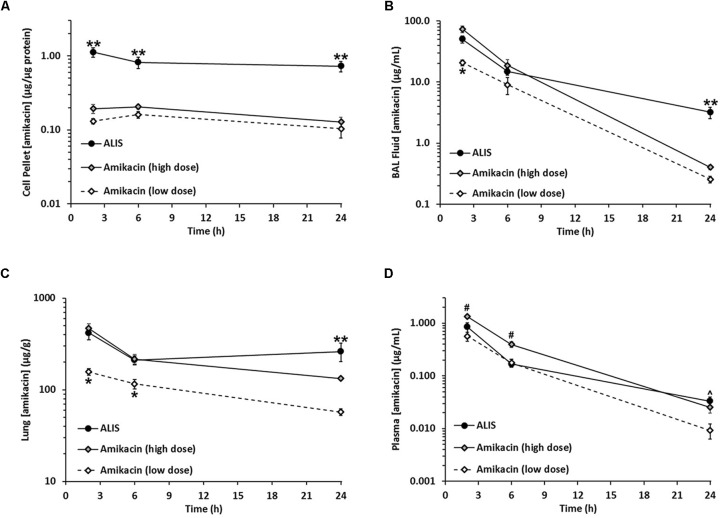
Amikacin liposome inhalation suspension (ALIS) enhanced amikacin uptake into macrophages and retained more amikacin in airways and lung tissue. Rats were administered either ALIS or free amikacin by nebulization and BAL cells (representing >80% macrophages) **(A)**, BAL fluid **(B)**, lungs **(C)**, and plasma **(D)** were collected at 2, 6, or 24 h after the end of nebulization. Amikacin concentrations were quantified by LC-MS/MS. The delivered and deposited doses for each group are presented in **Table 2**. ^∗^*P* < 0.05 vs. both high-dose amikacin and ALIS groups. ^∗∗^*P* < 0.05 vs. both low and high-dose amikacin groups. ^#^*P* < 0.05 vs. both ALIS and low-dose amikacin groups. ˆ*P* < 0.05 vs. the low-dose amikacin group. *n* = 8 rats per group.

We collected cells in the BAL fluid after inhalation administration of ALIS or free amikacin and measured amikacin concentrations in the cell pellets. The amount of protein in the cell pellets was not different between dosing groups at any timepoint (**Table 3**), indicating consistent collection of cells across the different dose groups. Cells harvested from the BAL fluid from rats dosed with ALIS exhibited 5- to 8-fold higher amikacin concentrations than BAL cells collected from rats dosed with low-dose free amikacin (**Figure 6A**). Additionally, the ALIS group contained 4- to 6-fold higher amikacin concentrations in the BAL cell pellet compared to the high-dose free amikacin group (**Figure 6A**), despite a twofold lower deposited dose in the ALIS group.

**Table 3:** Protein concentrations of cell pellets collected by bronchoalveolar lavage (BAL).

Timepoint	ALIS	Amikacin (low dose)	Amikacin (high dose)	*P*-value
2	79 ± 8	98 ± 10	72 ± 12	0.1957
6	90 ± 10	78 ± 7	91 ± 7	0.4570
24	108 ± 13	101 ± 19	115 ± 16	0.8320

*BAL cell pellet protein concentrations (μg/mL) were measured by BCA protein assay. *n* = 8 per timepoint in each group.*

We also quantified amikacin levels in BAL fluid (representing amikacin collected from airways) and found that samples from rats in the ALIS group had significantly higher amikacin concentrations at 2 h post-dose than BAL fluid samples from rats in the low-dose amikacin group (**Figure 6B**). Despite a higher dose deposited in the lungs, high-dose amikacin did not achieve significantly higher BAL fluid concentrations than ALIS (**Figure 6B**). At 24 h post-dose, the ALIS group exhibited significantly higher BAL fluid amikacin concentrations than both groups administered free amikacin (**Figure 6B**).

Following each BAL procedure, we excised and froze the lungs for measurement of tissue amikacin concentrations. Lung concentrations at 2 and 6 h post-dose in the ALIS group were significantly higher than those in the low-dose amikacin group (although the two groups achieved similar deposited doses) and equivalent to amikacin concentrations in the high-dose amikacin group (despite a ∼2-fold lower deposited dose with ALIS; **Figure 6C**). At 24 h post-dose, lung concentrations in the ALIS group were significantly higher than both low- and high-dose amikacin (**Figure 6C**).

Plasma amikacin concentrations were not significantly different between the ALIS and low dose amikacin groups at 2 and 6 h post-dose (**Figure 6D**). Plasma concentrations were significantly higher in the ALIS group relative to the low-dose amikacin group, but not the high-dose group, at 24 h post-dose (**Figure 6D**).

Because intravenous amikacin is a common treatment for refractory NTM infections, we also evaluated the distribution of amikacin into pulmonary macrophages, airways, and lung tissue after an intravenous bolus dose. The 100 mg/kg dose given intravenously was comparable to the 96 mg/kg amikacin dose delivered by ALIS nebulization. The mean peak plasma concentration at 0.25 h post-dose was 337 μg/mL and the mean lung concentration at that timepoint was 94 μg/g, approximately 10-fold lower than the 977 μg/g deposited dosed achieved with nebulized ALIS (**Table 2**). In pulmonary macrophages, amikacin concentrations were less than 0.004 μg/μg protein throughout the 24-h time course and the area under the curve (AUC) was 274-fold lower than the macrophage AUC following ALIS inhalation (**Table 4**). Similarly, AUCs in BAL fluid and lung tissue were 69- and 42-fold lower, respectively, after intravenous dosing compared with inhalation dosing of ALIS (**Table 4**).

**Table 4:** Summary of macrophage, airway, lung tissue, and plasma exposures.

				Fold difference	
	IV Amikacin	Inh Amikacin	ALIS	ALIS vs. IV Amikacin	ALIS vs. Inh Amikacin
Macrophages	0.1	2.9	17.8	274.2	6.1
Airways	4.2	142.5	292.6	69.5	2.1
Lung tissue	162.1	2771.0	6917.0	42.7	2.5
Plasma	22.6	3.1	3.8	0.2	1.2

*Values represent area under the curve (AUC) calculated for each group using the mean concentrations at each timepoint. AUC_2-24_ was calculated for macrophage, airway, and plasma exposures, whereas AUC_0-24_ was calculated for lung exposure.*

## Discussion

Amikacin liposome inhalation suspension was designed to provide efficient nebulization delivery of liposomes to the lungs, distribute to both central and peripheral regions of the lungs, and penetrate into biofilms and macrophages to reach the sites of infection. Li et al. (2008) showed that ALIS nebulization provides a consistent combination of encapsulated and free amikacin and forms aerosol droplets within the respirable range, and Meers et al. (2008) and Malinin et al. (2016) demonstrated that ALIS distributes well throughout different lobes of the lungs and into peripheral airways. The data presented herein clearly establish that ALIS can penetrate M. avium biofilms and into macrophages.

Amikacin liposome inhalation suspension effectively penetrated M. avium biofilms to reduce the number of viable bacteria; this was true for 100% ALIS as well as 70% ALIS/30% free amikacin (representing nebulized ALIS). In vivo, ALIS retained more amikacin at 24 h in airways and lung tissue compared to free amikacin, which would increase the duration of amikacin exposure at the sites of biofilm infections within mucus and alveolar walls.

Additionally, ALIS significantly enhanced amikacin uptake into macrophages compared with free amikacin. Using flow cytometry to quantify TAMRA fluorescence in macrophages, we found that the liposomal formulation enhanced amikacin uptake following 4- and 24-h incubations. Enhanced uptake with ALIS relative to free amikacin was also evident by fluorescence microscopy. These in vitro studies relied on the use of fluorescent amikacin, but we confirmed in a pilot study that TAMRA-conjugation did not alter uptake of free amikacin into macrophages. These data demonstrate that ALIS significantly increased amikacin uptake into human macrophages by ∼4-fold relative to free amikacin over a range of concentrations. The finding of improved uptake into macrophages in vitro is consistent with published data showing that ALIS kills intracellular M. avium infections in macrophages better than free amikacin (Rose et al., 2014).

Pulmonary macrophages from rats dosed with ALIS exhibited 5- to 8-fold higher amikacin levels compared with cells from animals given low-dose free amikacin, although the deposited dose immediately after nebulization was not significantly different between the groups. Additionally, the ALIS group exhibited significantly higher amikacin concentrations in BAL fluid and lung tissue relative to the low-dose amikacin group. These results show that the ALIS formulation retains amikacin within macrophages, airways, and lung tissue significantly better than free amikacin. Furthermore, high-dose amikacin deposited twofold more amikacin in the lungs immediately post-dose compared to ALIS, but failed to produce macrophage amikacin concentrations equaling those in the ALIS treatment group (4- to 6-fold lower with high-dose amikacin vs. ALIS). Amikacin levels in the bronchoalveolar space and lung tissue declined more over 24 h after administration of high-dose free amikacin relative to ALIS, indicating that free amikacin passes out of the lungs faster than ALIS. Taken together, these data provide strong evidence that inhaled ALIS can deliver more amikacin into macrophages in vivo than free drug. Importantly, simply depositing more free amikacin into the lungs failed to match the macrophage amikacin concentrations achieved with ALIS.

Intravenous amikacin is commonly used in patients with severe or refractory NTM-LD, but we found that free amikacin penetrated very poorly into lung tissue, airways, and pulmonary macrophages after intravenous administration. Compared to intravenous amikacin, ALIS achieved ∼42-, ∼69-, and ∼274-fold higher amikacin exposures in lung tissue, airways, and pulmonary macrophages, respectively, with ∼5-fold lower plasma exposure. Although intravenous amikacin is the current standard of care, ALIS delivered more antibiotic to sites relevant for NTM lung infections with substantially lower systemic exposure.

Amikacin liposome inhalation suspension has been shown to effectively treat NTM-LD in both preclinical studies and clinical trials. In mice with pulmonary M. avium infections, inhalation administration of ALIS lowered viable mycobacteria in the lungs by more than 2 log units (Rose et al., 2014). A Phase II trial of patients with refractory NTM-LD demonstrated that ALIS increased the proportion of patients who achieved negative sputum cultures compared with placebo, and the time to first negative sputum culture was shorter with ALIS treatment versus placebo (Olivier et al., 2017). Based on top-line results, an on-going Phase III study met the primary endpoint by demonstrating that the addition of ALIS to guideline based therapy eliminated evidence of NTM-LD caused by MAC in sputum by month 6 in a greater proportion of patients than guideline based therapy alone (Insmed, 2018).

Overall, the data presented herein demonstrate that ALIS delivers amikacin to pulmonary macrophages, airways, and lung tissue better than free amikacin given by either inhalation or intravenous administration. Simply delivering more free amikacin to the lungs by nebulization failed to match the macrophage uptake or duration of exposure achieved by ALIS, indicating the benefit of the liposomal formulation. This mechanism of improved delivery into pulmonary macrophages and retention within airways and lung tissue has been shown to effectively treat refractory NTM-LD in clinical trials and represents a promising new therapy for patients.

## Author Contributions

FL, JZ, SR, WP, and KD contributed conception and design of the studies. KD performed the statistical analyses. FL and KD wrote the manuscript. JZ, DC, SR, MN, JJ, JT, and TH performed the experiments. All authors contributed to manuscript revision, and then read and approved the submitted version.

## Conflict of Interest Statement

Authors JZ, FL, SR, DC, JT, TH, MN, WP, and KD were employed by Insmed Incorporated. The other author JJ declares no competing interests. The funder, Insmed Incorporated, had no role in study design, data collection and analysis, but was involved in the decision to publish the manuscript.
